# Clinical and Demographic Characteristics of Pyruvate Kinase Deficiency Patients: A Comprehensive Case Series Analysis

**DOI:** 10.7759/cureus.60035

**Published:** 2024-05-10

**Authors:** Abdulrahman Nasiri, Alfadil Haroon, Hazzaa Alzahrani

**Affiliations:** 1 Department of Internal Medicine, Imam Mohammad Ibn Saud Islamic University, Riyadh, SAU; 2 Section of Hematology, King Faisal Specialist Hospital and Research Centre, Riyadh, SAU

**Keywords:** blood transfusions, hereditary diseases, total splenectomy, autosomal recessive disorders, pyruvate kinase deficiency

## Abstract

Introduction

Pyruvate kinase deficiency (PKD) is a rare autosomal recessive disorder characterized by mutations in the PKLR gene, causing impaired glycolysis in red blood cells and leading to diverse clinical manifestations. The prevalence of PKD in Saudi Arabia remains understudied, particularly in the context of consanguinity and non-specialized medical facilities.

Methods

We conducted a retrospective analysis of seven PKD patients of Arab ethnicity, focusing on demographics, medical history, clinical features, laboratory results, treatments, and outcomes.

Results

Our patient cohort comprised five males and two females, aged 10 to 38 years, of Arab ethnicity. Consanguinity was prevalent, and hereditary connections were identified in five patients. PKD exhibited varying clinical presentations, with early-onset symptoms including neonatal jaundice and symptomatic anemia. One patient experienced severe hepatic disease progression leading to multiorgan failure. Blood transfusions were universally required, indicating the severity of the disorder. Anemia severity varied among patients, with diverse hematological irregularities. Splenectomy was performed for most patients, improving hemoglobin levels and transfusion needs in some cases. Iron chelation was administered, although iron overload persisted. Thrombocytosis and venous thromboembolism were observed post splenectomy. Jaundice and gallstones were common, leading to cholecystectomy. Laboratory findings remained consistent, with heightened reticulocyte counts and altered enzyme levels.

Discussion

PKD is a rare disorder characterized by diverse clinical manifestations. Prevalence estimation is complex due to various factors, and its diagnosis is challenged by clinical similarities with other disorders. Our cohort exhibited a spectrum of complications, highlighting the necessity for tailored interventions. Iron overload remained a concern, necessitating continuous monitoring. Although endocrine disorders and osteoporosis were absent in our cohort, vigilance is essential due to the disease's progressive nature. Genetic factors were prominent, supporting the genetic basis of PKD. Splenectomy improved anemia but had a limited impact on gallstones. Iron overload management and bone health remain crucial considerations.

Conclusion

This study offers comprehensive insights into the clinical and demographic characteristics of PKD patients, illustrating the complex nature of the disorder. The findings underscore the need for personalized management strategies and vigilant monitoring to address the diverse clinical manifestations and challenges associated with PKD.

## Introduction

Pyruvate kinase deficiency (PKD) is a rare, autosomal recessive disorder characterized by mutations in the PKLR gene, leading to impaired glycolysis in red blood cells. The enzyme deficiency affects the RBC function and leads to a wide range of clinical manifestations, necessitating personalized management. The prevalence of PKD in Saudi Arabia has not been extensively researched. This is particularly notable in a nation where consanguinity is common, and the overall prevalence of congenital hemolytic anemia presents a unique challenge in identifying specific disorders, especially within non-specialized medical facilities.

In this paper, we are reporting case series to provide an in-depth examination of clinical and demographic characteristics of PKD patients, shedding light on its heterogeneity.

## Materials and methods

Study design

This retrospective case series aimed to provide a comprehensive analysis of PKD among Arab ethnicity patients under our care. The study included seven individuals with PKD, with data collection and analysis spanning a specified period.

Data collection

Data collection involved a meticulous review of patient records and medical histories, encompassing demographic information, detailed medical history, clinical features, laboratory results, treatment regimens, and clinical outcomes. The data extraction process adhered to strict privacy and ethical considerations.

Study population

The study cohort comprised seven individuals diagnosed with PKD. Inclusion criteria encompassed patients of Arab ethnicity with confirmed PKD diagnosis based on clinical and laboratory assessments.

Demographic information

Demographic information, such as age, gender, and family background, was obtained from the patient records. Additionally, consanguinity within families and any family history of PKD were documented.

Clinical assessment

A comprehensive clinical assessment was conducted to evaluate the diverse range of PKD manifestations, including symptoms onset, disease progression, and complications. Special attention was given to the presentation of neonatal jaundice and symptomatic anemia, as these are common early indicators of the disorder.

Laboratory evaluation

Laboratory investigations were performed to assess key markers associated with PKD. This included measuring pyruvate kinase (PK) activity in all patients and genetic testing in a subset of patients to identify pathogenic variants in the PKLR gene.

Treatment regimens

The study examined the treatment regimens administered to the patients, which typically included therapies such as folic acid supplementation, iron chelation, prophylactic antibiotics, and aspirin. The rationale behind treatment choices and their effectiveness were considered.

Clinical outcomes

Clinical outcomes, including hemoglobin levels, hematological irregularities, complications such as thrombocytosis, venous thromboembolism, and gallstones, as well as the impact of splenectomy on anemia and transfusion dependency, were assessed and documented.

Iron overload management

The study addressed the management of iron overload, a common concern in PKD patients, through regular monitoring of ferritin levels and the use of magnetic resonance imaging (MRI) for iron level assessment.

Statistical analysis

Descriptive statistics were employed to summarize demographic and clinical data. Continuous variables were presented as means or ranges, while categorical variables were summarized using percentages.

## Results

The group of patients under study consisted of five males and two females, whose ages ranged from 10 to 38 years. All individuals in this cohort identified as Arabs. Baseline demographic information is presented in Table 1.

**Table 1:** Demographic features and common complications of enrolled patients.

Characteristics	Number	%
Sex		
Male	5	71.5%
Female	2	28.5%
Arab ethnicity	7	100%
Consanguinity	6	85.7%
Splenectomized	6	85.7%
Gallstones	6	85.7%
Thrombocytosis	5	71.5%
Thrombosis	1	14.28%
Currently receiving regular transfusions	3	42.8%

Regarding their family backgrounds, all patients except one had consanguinity in their families. Among the patients, five had a family member with PKD, indicating a hereditary connection.

PKD, a condition that endures throughout one's life, presents a diverse range of clinical manifestations, spanning from hydrops fetalis to individuals who show compensatory mechanisms with minimal symptoms and hemolysis.

In our observed patient group, the onset of symptoms was consistent across the board, manifesting in early ages as either neonatal jaundice or symptomatic anemia.

One patient developed severe hepatic disease with evolution to liver failure, followed by multiorgan failure and death.

**Figure 1:**
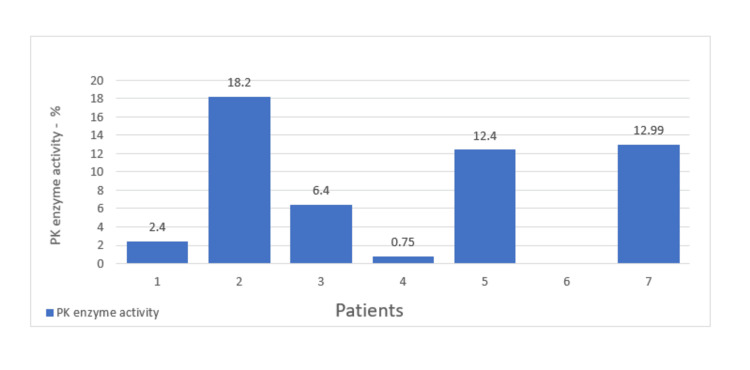
Pyruvate kinase (PK) enzyme activity.

The PK activity was done on all patients, and all were below 19% (range: 0.75-18.2). Genetic testing was done for one patient and showed pathogenic variants in PKLR (Figure 1).

The necessity for blood transfusions was universal among all patients, illustrating the severe clinical nature of their condition.

Anemia severity exhibited a spectrum, with 66.66% of patients experiencing mild to moderate anemia and 33.33% displaying severe anemia.

Hemoglobin levels showed considerable variation, ranging from 6.5 to 9 g/dL. Hematological irregularities included anisopoikilocytosis, thrombocytosis, and leucoerythroblastic blood patterns, reflecting the intricate and diverse nature of PKD manifestations.

There was no report of myelodysplastic syndrome, leg ulcers, or pulmonary hypertension.

The majority of patients in this study underwent splenectomy as part of their treatment strategy, with the exception of one patient who did not due to death. The primary objectives of this intervention were to improve baseline anemia, reduce dependence on transfusions, and enhance overall patient quality of life.

Following splenectomy, three patients had a rise in the hemoglobin level and a decrease in the need for transfusion support, while the other patients were not able to discontinue transfusions following the surgery.

As a consequence of the disease, all patients received iron chelation as part of their treatment regimen, effectively addressing iron overload, a common concern in PKD. The mean ferritin was 3325.9 ng/mL for the overall study cohort. However, iron overload persisted in all cases, necessitating continuous monitoring and management.

Thrombocytosis was evident in five patients post splenectomy and one patient developed venous thromboembolism in the form of bilateral pulmonary embolism.

Jaundice was a common clinical and biochemical observation among all patients and was complicated by the presence of gallstones, a frequent complication of PK deficiency for which cholecystectomy was performed on six patients.

Laboratory findings remained consistent across all patients, characterized by heightened reticulocyte counts, elevated lactate dehydrogenase levels and indirect hyperbilirubinemia, and decreased haptoglobin levels.

Commonly prescribed medications in our cohort included folic acid, iron chelation, prophylactic antibiotics, and aspirin.

## Discussion

**Figure 2:**
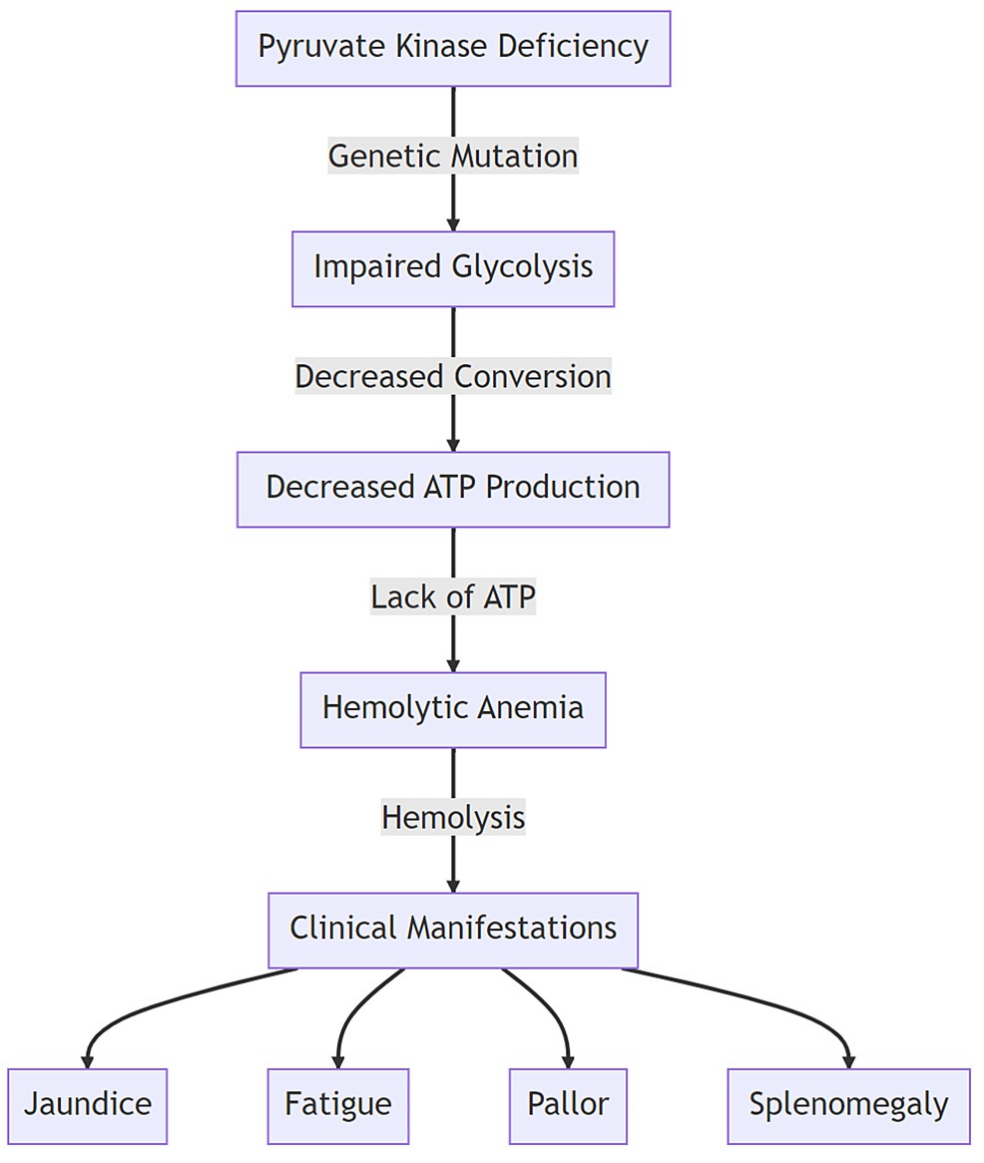
The enzyme deficiency affects the RBC function and leads to a wide range of clinical manifestations. ATP: adenosine triphosphate. Original work of the authors.

PKD is a rare, autosomal recessive enzymopathy disorder characterized by mutations in the PKLR gene (PK, liver, and red cell isoform), which is located on chromosome 1q21 [1]. The PK enzyme is an integral part of maintaining the energy production in the red blood cells (RBCs), as mature RBCs lack the presence of mitochondria and rely on glycolysis. The lack of the enzyme leads to chronic hereditary non-spherocytic hemolytic anemia (Figure 2) [2].

The disease prevalence is largely unknown with different reports from around the world estimating an occurrence between 3.2 and 8.5 per million in the western part of the world [3].

PKD prevalence in Saudi Arabia is still an area of active research. An early study by Abu-Melha et al. collected 513 random samples and found that 3.2% were deficient; however, its clinical significance is unknown [4]. Another study was done earlier to estimate the prevalence of PKD and found partial deficiency; however, no complete deficiency was identified [5]. To date, the incidence of the disease is unknown.

The disease prevalence estimation is challenged by the different PK activity assay methods and reporting methods, as studies have focused on cases that were diagnosed based on clinical misdiagnosis due to similarity to other RBC disorders and heterogeneous clinical presentation [3,6].

PKD is a lifelong disorder and has heterogeneous clinical manifestations ranging from hydrops fetalis to asymptomatic compensating individuals with hemolysis. The patients in our cohort all manifested at an early age with either neonatal jaundice or symptomatic anemia; however, its absence does not exclude PKD [7].

Other manifestations might include liver failure and multiorgan failure, which was encountered with one patient and was reported in the literature [8,9].

With advancing age, patients may manifest a spectrum of disease-related complications. Notably, these include jaundice and gallstone formation, accompanied by systemic manifestations such as iron overload. Anemia of varying severity is prevalent, alongside thrombocytosis, which may precipitate thrombotic events. Furthermore, these individuals are at an increased risk for pulmonary hypertension and diverse endocrinopathies. Osteoporosis, potentially leading to fractures, along with extramedullary hematopoiesis and lower extremity ulcers, also contribute to the multifaceted clinical challenges encountered in managing this disease [10].

The prevalence of these complications varies among patients, necessitating careful monitoring to identify the subtle signs and symptoms associated with these conditions. It is important to note that none of the patients in our cohort exhibited endocrine disorders, pulmonary hypertension, or osteoporosis. This absence of certain complications is likely attributable to the relatively young age of the patients included in our study [11].

Consanguinity is common in certain parts of the world, including the Arab world and particularly Saudi Arabia, and its association with the prevalence of PKD is complex. It was found in all the patients except one, with five patients having an affected family member with PKD, solidifying its genetic basis [12].

The patients in our cohort were all in need of frequent transfusions as most of the patients with PKD underwent splenectomy, which was successful in three patients. The data from the literature are inclining toward total splenectomy as partial resection results were not optimal [10].

Transfusions should be administered based on a clinical assessment that considers factors such as quality of life, growth, and symptoms, rather than relying solely on hemoglobin levels, as it carries the risk of transfusion-related complications. Iron overload occurs regardless of the transfusion status; however, it is augmented on the patient receiving blood [13].

Splenectomized patients have increased platelet counts and are prone to have venous thromboembolism compared to non-splenectomized patients or those who were splenectomized for non-hematological reasons. Despite being a significant potential complication, its occurrence is low, and splenectomy improves anemia and reduces the transfusion frequency [14].

Splenectomy did not show an impact on the formation of gallstones and the risk of developing pigmented stones persists. The need for cholecystectomy is inevitable in most patients and cholecystectomy should be contemplated during splenectomy [10,15].

Iron overload is an unavoidable complication and chelating agents are a must. It is recommended to conduct annual monitoring of ferritin levels and utilize MRI for assessing iron levels [16].

While none of our patients exhibited osteopenia, it is important to note that individuals with PKD remain susceptible to its development. Therefore, attention to vitamin D and calcium consumption is advisable, considering baseline assessment during late adolescence or early adulthood, and monitoring through dual-energy X-ray absorptiometry scans should be considered [10].

To our knowledge, this is the first report of clinical manifestations of PKD patients from Saudi Arabia and potentially from the broader Arab region. However, the study is not without its limitations. It is a single-center study, which may affect the generalizability of the results across the region. Additionally, most of the patients did not undergo genetic studies, which limits our understanding of the genetic diversity and specific mutations associated with PKD in this population. Further research involving multiple centers and comprehensive genetic analysis is essential to overcome these limitations and provide a more detailed understanding of PKD across Saudi Arabia and the Arab region.

## Conclusions

This study provides valuable insights into the diverse and complex clinical manifestations of PKD. Our analysis of a cohort comprising both males and females of Arab ethnicity, spanning a wide age range, highlights the hereditary nature of the disorder, as evidenced by consanguinity and family history among patients. The findings emphasize the severe impact of PKD, necessitating universal blood transfusion and often requiring splenectomy to manage anemia and enhance quality of life.
